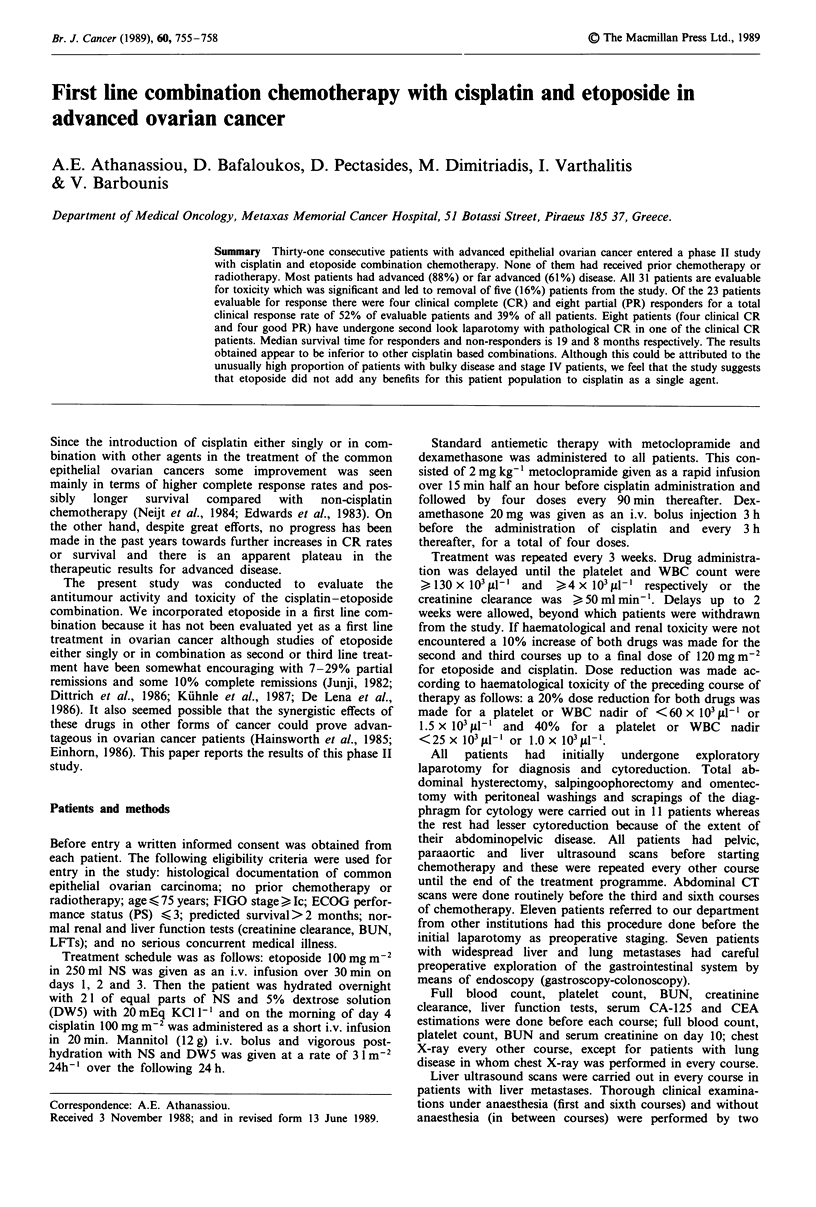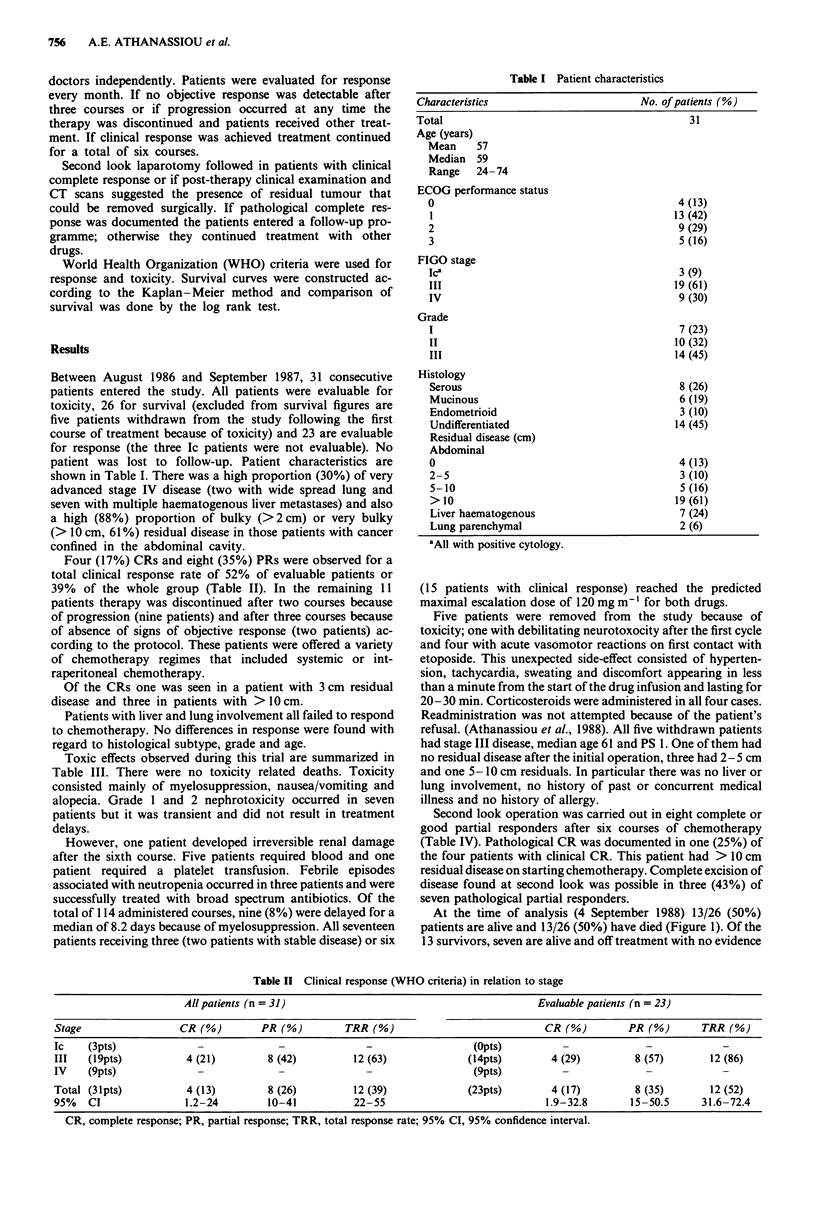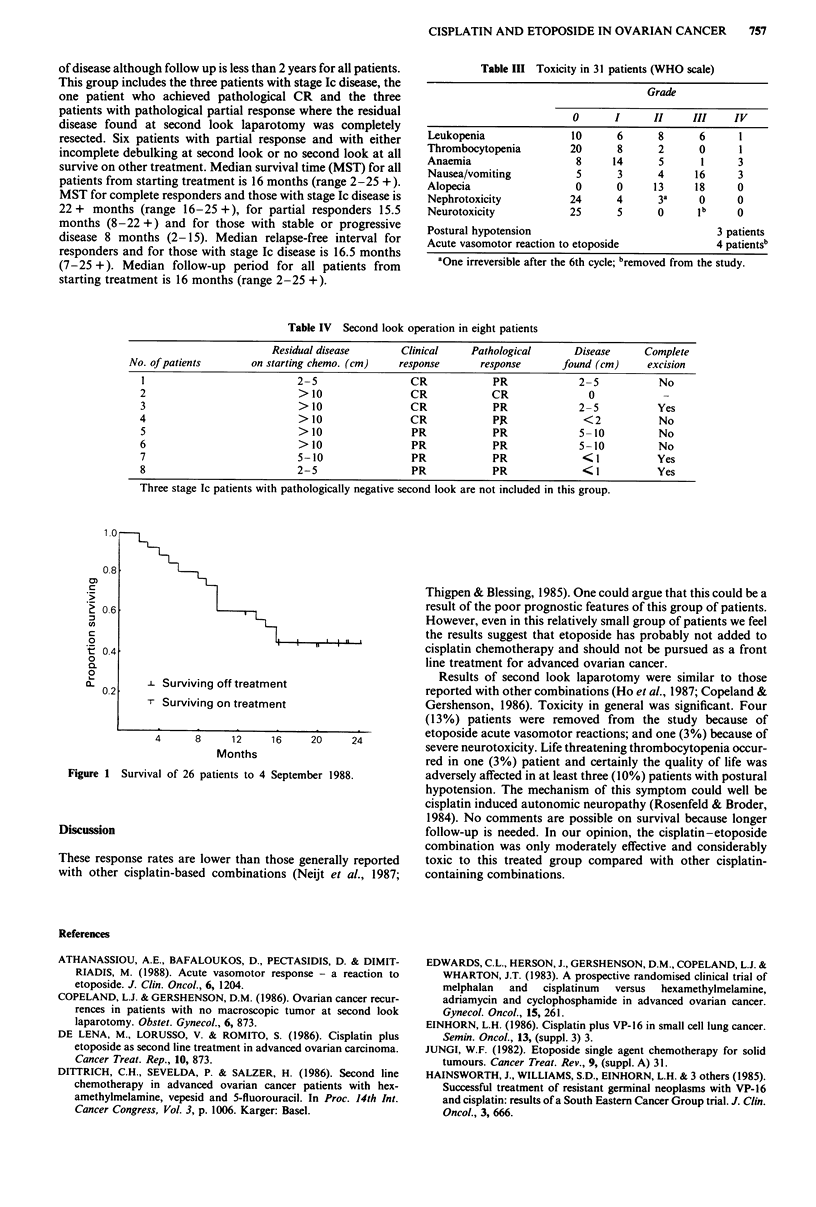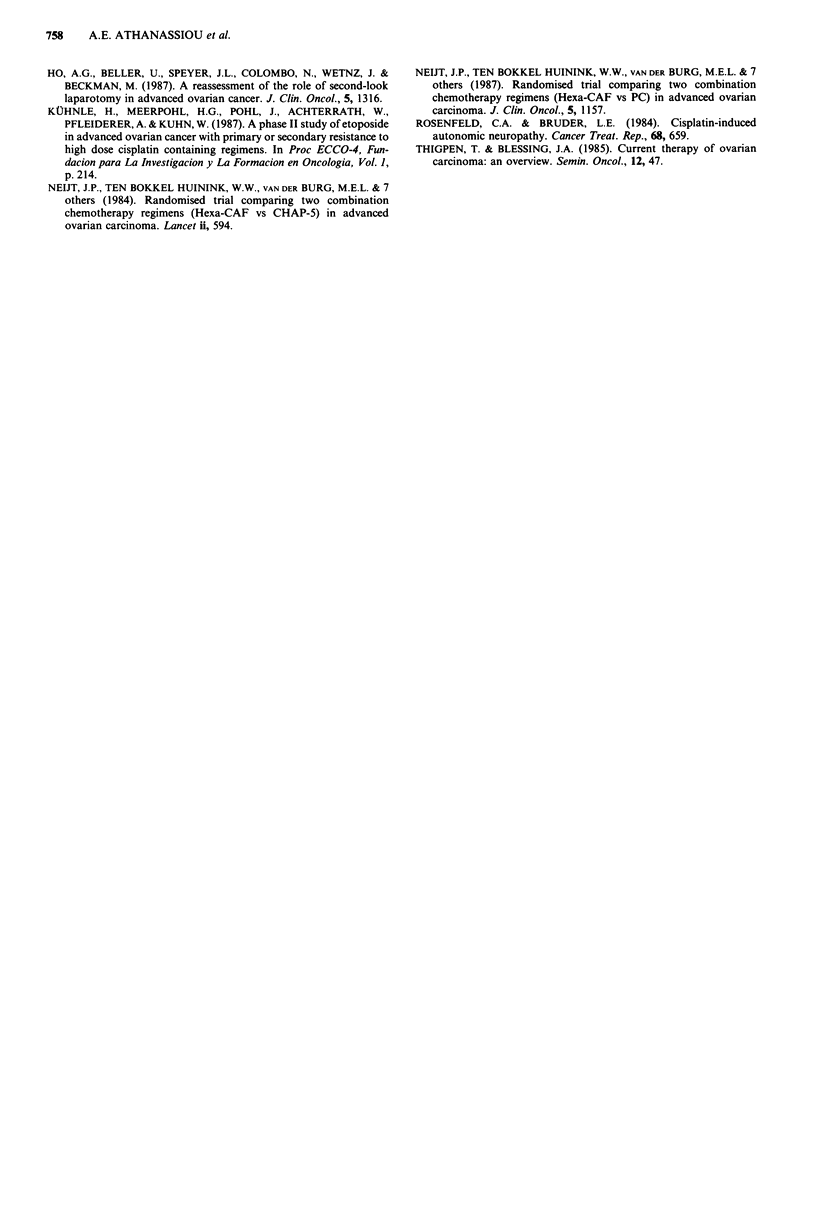# First line combination chemotherapy with cisplatin and etoposide in advanced ovarian cancer.

**DOI:** 10.1038/bjc.1989.353

**Published:** 1989-11

**Authors:** A. E. Athanassiou, D. Bafaloukos, D. Pectasides, M. Dimitriadis, I. Varthalitis, V. Barbounis

**Affiliations:** Department of Medical Oncology, Metaxas Memorial Cancer Hospital, Piraeus, Greece.

## Abstract

Thirty-one consecutive patients with advanced epithelial ovarian cancer entered a phase II study with cisplatin and etoposide combination chemotherapy. None of them had received prior chemotherapy or radiotherapy. Most patients had advanced (88%) or far advanced (61%) disease. All 31 patients are evaluable for toxicity which was significant and led to removal of five (16%) patients from the study. Of the 23 patients evaluable for response there were four clinical complete (CR) and eight partial (PR) responders for a total clinical response rate of 52% of evaluable patients and 39% of all patients. Eight patients (four clinical CR and four good PR) have undergone second look laparotomy with pathological CR in one of the clinical CR patients. Median survival time for responders and non-responders is 19 and 8 months respectively. The results obtained appear to be inferior to other cisplatin based combinations. Although this could be attributed to the unusually high proportion of patients with bulky disease and stage IV patients, we feel that the study suggests that etoposide did not add any benefits for this patient population to cisplatin as a single agent.


*Br. J. Cancer* (1989), **60**, 755–758 © The Macmillan Press Ltd., 1989

### First line combination chemotherapy with cisplatin and etoposide in advanced ovarian cancer

A.E. Athanassiou, D. Bafaloukos, D. Pectasides, M. Dimitriadis, I. Varthalitis & V. Barbounis

*Department of Medical Oncology, Metaxas Memorial Cancer Hospital, 51 Botassi Street, Piraeus 185 37, Greece.*

**Summary** Thirty-one consecutive patients with advanced epithelial ovarian cancer entered a phase II study with cisplatin and etoposide combination chemotherapy. None of them had received prior chemotherapy or radiotherapy. Most patients had advanced (88%) or far advanced (61%) disease. All 31 patients are evaluable for toxicity which was significant and led to removal of five (16%) patients from the study. Of the 23 patients evaluable for response there were four clinical complete (CR) and eight partial (PR) responders for a total clinical response rate of 52% of evaluable patients and 39% of all patients. Eight patients (four clinical CR and four good PR) have undergone second look laparotomy with pathological CR in one of the clinical CR patients. Median survival time for responders and non-responders is 19 and 8 months respectively. The results obtained appear to be inferior to other cisplatin based combinations. Although this could be attributed to the unusually high proportion of patients with bulky disease and stage IV patients, we feel that the study suggests that etoposide did not add any benefits for this patient population to cisplatin as a single agent.

Since the introduction of cisplatin either singly or in combination with other agents in the treatment of the common epithelial ovarian cancers some improvement was seen mainly in terms of higher complete response rates and possibly longer survival compared with non-cisplatin chemotherapy (Neijt *et al.*, 1984; Edwards *et al.*, 1983). On the other hand, despite great efforts, no progress has been made in the past years towards further increases in CR rates or survival and there is an apparent plateau in the therapeutic results for advanced disease.

The present study was conducted to evaluate the antitumour activity and toxicity of the cisplatin–etoposide combination. We incorporated etoposide in a first line combination because it has not been evaluated yet as a first line treatment in ovarian cancer although studies of etoposide either singly or in combination as second or third line treatment have been somewhat encouraging with 7–29% partial remissions and some 10% complete remissions (Junji, 1982; Dittrich *et al.*, 1986; Kühnle *et al.*, 1987; De Lena *et al.*, 1986). It also seemed possible that the synergistic effects of these drugs in other forms of cancer could prove advantageous in ovarian cancer patients (Hainsworth *et al.*, 1985; Einhorn, 1986). This paper reports the results of this phase II study.

### Patients and methods

Before entry a written informed consent was obtained from each patient. The following eligibility criteria were used for entry in the study: histological documentation of common epithelial ovarian carcinoma; no prior chemotherapy or radiotherapy; age $\leqslant$ 75 years; FIGO stage $\geqslant$ Ic; ECOG performance status (PS) $\leqslant 3$; predicted survival $>$ 2 months; normal renal and liver function tests (creatinine clearance, BUN, LFTs); and no serious concurrent medical illness.

Treatment schedule was as follows: etoposide 100 mg m$^{-2}$ in 250 ml NS was given as an i.v. infusion over 30 min on days 1, 2 and 3. Then the patient was hydrated overnight with 2 l of equal parts of NS and 5% dextrose solution (DW5) with 20 mEq KCl l$^{-1}$ and on the morning of day 4 cisplatin 100 mg m$^{-2}$ was administered as a short i.v. infusion in 20 min. Mannitol (12 g) i.v. bolus and vigorous post-hydration with NS and DW5 was given at a rate of 3 l m$^{-2}$ 24h$^{-1}$ over the following 24 h.

Standard antiemetic therapy with metoclopramide and dexamethasone was administered to all patients. This consisted of 2 mg kg$^{-1}$ metoclopramide given as a rapid infusion over 15 min half an hour before cisplatin administration and followed by four doses every 90 min thereafter. Dexamethasone 20 mg was given as an i.v. bolus injection 3 h before the administration of cisplatin and every 3 h thereafter, for a total of four doses.

Treatment was repeated every 3 weeks. Drug administration was delayed until the platelet and WBC count were $\geqslant 130 \times 10^3\,\mu l^{-1}$ and $\geqslant 4 \times 10^3\,\mu l^{-1}$ respectively or the creatinine clearance was $\geqslant 50$ ml min$^{-1}$. Delays up to 2 weeks were allowed, beyond which patients were withdrawn from the study. If haematological and renal toxicity were not encountered a 10% increase of both drugs was made for the second and third courses up to a final dose of 120 mg m$^{-2}$ for etoposide and cisplatin. Dose reduction was made according to haematological toxicity of the preceding course of therapy as follows: a 20% dose reduction for both drugs was made for a platelet or WBC nadir of $<60 \times 10^3\,\mu l^{-1}$ or $1.5 \times 10^3\,\mu l^{-1}$ and 40% for a platelet or WBC nadir $<25 \times 10^3\,\mu l^{-1}$ or $1.0 \times 10^3\,\mu l^{-1}$.

All patients had initially undergone exploratory laparotomy for diagnosis and cytoreduction. Total abdominal hysterectomy, salpingoophorectomy and omentectomy with peritoneal washings and scrapings of the diaphragm for cytology were carried out in 11 patients whereas the rest had lesser cytoreduction because of the extent of their abdominopelvic disease. All patients had pelvic, paraaortic and liver ultrasound scans before starting chemotherapy and these were repeated every other course until the end of the treatment programme. Abdominal CT scans were done routinely before the third and sixth courses of chemotherapy. Eleven patients referred to our department from other institutions had this procedure done before the initial laparotomy as preoperative staging. Seven patients with widespread liver and lung metastases had careful preoperative exploration of the gastrointestinal system by means of endoscopy (gastroscopy-colonoscopy).

Full blood count, platelet count, BUN, creatinine clearance, liver function tests, serum CA-125 and CEA estimations were done before each course; full blood count, platelet count, BUN and serum creatinine on day 10; chest X-ray every other course, except for patients with lung disease in whom chest X-ray was performed in every course.

Liver ultrasound scans were carried out in every course in patients with liver metastases. Thorough clinical examinations under anaesthesia (first and sixth courses) and without anaesthesia (in between courses) were performed by two

Correspondence: A.E. Athanassiou.
Received 3 November 1988; and in revised form 13 June 1989.

doctors independently. Patients were evaluated for response every month. If no objective response was detectable after three courses or if progression occurred at any time the therapy was discontinued and patients received other treatment. If clinical response was achieved treatment continued for a total of six courses.

Second look laparotomy followed in patients with clinical complete response or if post-therapy clinical examination and CT scans suggested the presence of residual tumour that could be removed surgically. If pathological complete response was documented the patients entered a follow-up programme; otherwise they continued treatment with other drugs.

World Health Organization (WHO) criteria were used for response and toxicity. Survival curves were constructed according to the Kaplan-Meier method and comparison of survival was done by the log rank test.

### Results

Between August 1986 and September 1987, 31 consecutive patients entered the study. All patients were evaluable for toxicity, 26 for survival (excluded from survival figures are five patients withdrawn from the study following the first course of treatment because of toxicity) and 23 are evaluable for response (the three Ic patients were not evaluable). No patient was lost to follow-up. Patient characteristics are shown in Table I. There was a high proportion (30%) of very advanced stage IV disease (two with wide spread lung and seven with multiple haematogenous liver metastases) and also a high (88%) proportion of bulky (> 2 cm) or very bulky (> 10 cm, 61%) residual disease in those patients with cancer confined in the abdominal cavity.

Four (17%) CRs and eight (35%) PRs were observed for a total clinical response rate of 52% of evaluable patients or 39% of the whole group (Table II). In the remaining 11 patients therapy was discontinued after two courses because of progression (nine patients) and after three courses because of absence of signs of objective response (two patients) according to the protocol. These patients were offered a variety of chemotherapy regimes that included systemic or intraperitoneal chemotherapy.

Of the CRs one was seen in a patient with 3 cm residual disease and three in patients with > 10 cm.

Patients with liver and lung involvement all failed to respond to chemotherapy. No differences in response were found with regard to histological subtype, grade and age.

Toxic effects observed during this trial are summarized in Table III. There were no toxicity related deaths. Toxicity consisted mainly of myelosuppression, nausea/vomiting and alopecia. Grade 1 and 2 nephrotoxicity occurred in seven patients but it was transient and did not result in treatment delays.

However, one patient developed irreversible renal damage after the sixth course. Five patients required blood and one patient required a platelet transfusion. Febrile episodes associated with neutropenia occurred in three patients and were successfully treated with broad spectrum antibiotics. Of the total of 114 administered courses, nine (8%) were delayed for a median of 8.2 days because of myelosuppression. All seventeen patients receiving three (two patients with stable disease) or six (15 patients with clinical response) reached the predicted maximal escalation dose of 120 mg $m^{-1}$ for both drugs.

**Table I** Patient characteristics

| *Characteristics* | *No. of patients (%)* |
|---|---|
| Total | 31 |
| Age (years) | |
| Mean 57 | |
| Median 59 | |
| Range 24-74 | |
| ECOG performance status | |
| 0 | 4 (13) |
| 1 | 13 (42) |
| 2 | 9 (29) |
| 3 | 5 (16) |
| FIGO stage | |
| Ic[a] | 3 (9) |
| III | 19 (61) |
| IV | 9 (30) |
| Grade | |
| I | 7 (23) |
| II | 10 (32) |
| III | 14 (45) |
| Histology | |
| Serous | 8 (26) |
| Mucinous | 6 (19) |
| Endometrioid | 3 (10) |
| Undifferentiated | 14 (45) |
| Residual disease (cm) | |
| Abdominal | |
| 0 | 4 (13) |
| 2-5 | 3 (10) |
| 5-10 | 5 (16) |
| > 10 | 19 (61) |
| Liver haematogenous | 7 (24) |
| Lung parenchymal | 2 (6) |

[a]All with positive cytology.

Five patients were removed from the study because of toxicity; one with debilitating neurotoxocity after the first cycle and four with acute vasomotor reactions on first contact with etoposide. This unexpected side-effect consisted of hypertension, tachycardia, sweating and discomfort appearing in less than a minute from the start of the drug infusion and lasting for 20-30 min. Corticosteroids were administered in all four cases. Readministration was not attempted because of the patient's refusal. (Athanassiou *et al.*, 1988). All five withdrawn patients had stage III disease, median age 61 and PS 1. One of them had no residual disease after the initial operation, three had 2-5 cm and one 5-10 cm residuals. In particular there was no liver or lung involvement, no history of past or concurrent medical illness and no history of allergy.

Second look operation was carried out in eight complete or good partial responders after six courses of chemotherapy (Table IV). Pathological CR was documented in one (25%) of the four patients with clinical CR. This patient had > 10 cm residual disease on starting chemotherapy. Complete excision of disease found at second look was possible in three (43%) of seven pathological partial responders.

At the time of analysis (4 September 1988) 13/26 (50%) patients are alive and 13/26 (50%) have died (Figure 1). Of the 13 survivors, seven are alive and off treatment with no evidence

**Table II** Clinical response (WHO criteria) in relation to stage

| | | *All patients* (n = *31*) | | | *Evaluable patients* (n = *23*) | | | |
|---|---|---|---|---|---|---|---|---|
| *Stage* | | *CR (%)* | *PR (%)* | *TRR (%)* | | *CR (%)* | *PR (%)* | *TRR (%)* |
| Ic | (3pts) | – | – | – | (0pts) | – | – | – |
| III | (19pts) | 4 (21) | 8 (42) | 12 (63) | (14pts) | 4 (29) | 8 (57) | 12 (86) |
| IV | (9pts) | – | – | – | (9pts) | – | – | – |
| Total | (31pts) | 4 (13) | 8 (26) | 12 (39) | (23pts) | 4 (17) | 8 (35) | 12 (52) |
| 95% | CI | 1.2-24 | 10-41 | 22-55 | | 1.9-32.8 | 15-50.5 | 31.6-72.4 |

CR, complete response; PR, partial response; TRR, total response rate; 95% CI, 95% confidence interval.

of disease although follow up is less than 2 years for all patients. This group includes the three patients with stage Ic disease, the one patient who achieved pathological CR and the three patients with pathological partial response where the residual disease found at second look laparotomy was completely resected. Six patients with partial response and with either incomplete debulking at second look or no second look at all survive on other treatment. Median survival time (MST) for all patients from starting treatment is 16 months (range 2–25 +). MST for complete responders and those with stage Ic disease is 22 + months (range 16–25 +), for partial responders 15.5 months (8–22 +) and for those with stable or progressive disease 8 months (2–15). Median relapse-free interval for responders and for those with stage Ic disease is 16.5 months (7–25 +). Median follow-up period for all patients from starting treatment is 16 months (range 2–25 +).

**Table III** Toxicity in 31 patients (WHO scale)

| | *Grade* | | | | |
|---|---|---|---|---|---|
| | *0* | *I* | *II* | *III* | *IV* |
| Leukopenia | 10 | 6 | 8 | 6 | 1 |
| Thrombocytopenia | 20 | 8 | 2 | 0 | 1 |
| Anaemia | 8 | 14 | 5 | 1 | 3 |
| Nausea/vomiting | 5 | 3 | 4 | 16 | 3 |
| Alopecia | 0 | 0 | 13 | 18 | 0 |
| Nephrotoxicity | 24 | 4 | 3[a] | 0 | 0 |
| Neurotoxicity | 25 | 5 | 0 | 1[b] | 0 |
| Postural hypotension | | | | | 3 patients |
| Acute vasomotor reaction to etoposide | | | | | 4 patients[b] |

[a]One irreversible after the 6th cycle; [b]removed from the study.

**Table IV** Second look operation in eight patients

| *No. of patients* | *Residual disease on starting chemo. (cm)* | *Clinical response* | *Pathological response* | *Disease found (cm)* | *Complete excision* |
|---|---|---|---|---|---|
| 1 | 2–5 | CR | PR | 2–5 | No |
| 2 | >10 | CR | CR | 0 | – |
| 3 | >10 | CR | PR | 2–5 | Yes |
| 4 | >10 | CR | PR | <2 | No |
| 5 | >10 | PR | PR | 5–10 | No |
| 6 | >10 | PR | PR | 5–10 | No |
| 7 | 5–10 | PR | PR | <1 | Yes |
| 8 | 2–5 | PR | PR | <1 | Yes |

Three stage Ic patients with pathologically negative second look are not included in this group.

**Figure 1** Survival of 26 patients to 4 September 1988.

### Discussion

These response rates are lower than those generally reported with other cisplatin-based combinations (Neijt *et al.*, 1987; Thigpen & Blessing, 1985). One could argue that this could be a result of the poor prognostic features of this group of patients. However, even in this relatively small group of patients we feel the results suggest that etoposide has probably not added to cisplatin chemotherapy and should not be pursued as a front line treatment for advanced ovarian cancer.

Results of second look laparotomy were similar to those reported with other combinations (Ho *et al.*, 1987; Copeland & Gershenson, 1986). Toxicity in general was significant. Four (13%) patients were removed from the study because of etoposide acute vasomotor reactions; and one (3%) because of severe neurotoxicity. Life threatening thrombocytopenia occurred in one (3%) patient and certainly the quality of life was adversely affected in at least three (10%) patients with postural hypotension. The mechanism of this symptom could well be cisplatin induced autonomic neuropathy (Rosenfeld & Broder, 1984). No comments are possible on survival because longer follow-up is needed. In our opinion, the cisplatin–etoposide combination was only moderately effective and considerably toxic to this treated group compared with other cisplatin-containing combinations.

### References

ATHANASSIOU, A.E., BAFALOUKOS, D., PECTASIDIS, D. & DIMITRIADIS, M. (1988). Acute vasomotor response – a reaction to etoposide. *J. Clin. Oncol.*, **6**, 1204.

COPELAND, L.J. & GERSHENSON, D.M. (1986). Ovarian cancer recurrences in patients with no macroscopic tumor at second look laparotomy. *Obstet. Gynecol.*, **6**, 873.

DE LENA, M., LORUSSO, V. & ROMITO, S. (1986). Cisplatin plus etoposide as second line treatment in advanced ovarian carcinoma. *Cancer Treat. Rep.*, **10**, 873.

DITTRICH, C.H., SEVELDA, P. & SALZER, H. (1986). Second line chemotherapy in advanced ovarian cancer patients with hexamethylmelamine, vepesid and 5-fluorouracil. In *Proc. 14th Int. Cancer Congress, Vol. 3*, p. 1006. Karger: Basel.

EDWARDS, C.L., HERSON, J., GERSHENSON, D.M., COPELAND, L.J. & WHARTON, J.T. (1983). A prospective randomised clinical trial of melphalan and cisplatinum versus hexamethylmelamine, adriamycin and cyclophosphamide in advanced ovarian cancer. *Gynecol. Oncol.*, **15**, 261.

EINHORN, L.H. (1986). Cisplatin plus VP-16 in small cell lung cancer. *Semin. Oncol.*, **13**, (suppl. 3) 3.

JUNGI, W.F. (1982). Etoposide single agent chemotherapy for solid tumours. *Cancer Treat. Rev.*, **9**, (suppl. A) 31.

HAINSWORTH, J., WILLIAMS, S.D., EINHORN, L.H. & 3 others (1985). Successful treatment of resistant germinal neoplasms with VP-16 and cisplatin: results of a South Eastern Cancer Group trial. *J. Clin. Oncol.*, **3**, 666.

HO, A.G., BELLER, U., SPEYER, J.L., COLOMBO, N., WETNZ, J. & BECKMAN, M. (1987). A reassessment of the role of second-look laparotomy in advanced ovarian cancer. *J. Clin. Oncol.*, **5**, 1316.

KÜHNLE, H., MEERPOHL, H.G., POHL, J., ACHTERRATH, W., PFLEIDERER, A. & KUHN, W. (1987). A phase II study of etoposide in advanced ovarian cancer with primary or secondary resistance to high dose cisplatin containing regimens. In *Proc ECCO-4, Fundacion para La Investigacion y La Formacion en Oncologia, Vol. 1*, p. 214.

NEIJT, J.P., TEN BOKKEL HUININK, W.W., VAN DER BURG, M.E.L. & 7 others (1984). Randomised trial comparing two combination chemotherapy regimens (Hexa-CAF vs CHAP-5) in advanced ovarian carcinoma. *Lancet* **ii**, 594.

NEIJT, J.P., TEN BOKKEL HUININK, W.W., VAN DER BURG, M.E.L. & 7 others (1987). Randomised trial comparing two combination chemotherapy regimens (Hexa-CAF vs PC) in advanced ovarian carcinoma. *J. Clin. Oncol.*, **5**, 1157.

ROSENFELD, C.A. & BRUDER, L.E. (1984). Cisplatin-induced autonomic neuropathy. *Cancer Treat. Rep.*, **68**, 659.

THIGPEN, T. & BLESSING, J.A. (1985). Current therapy of ovarian carcinoma: an overview. *Semin. Oncol.*, **12**, 47.